# A tick bite patient with fever and meningitis co-infected with *Rickettsia raoultii* and Tacheng tick virus 1: a case report

**DOI:** 10.1186/s12879-021-06877-z

**Published:** 2021-11-25

**Authors:** Yu Zhang, Liang Jiang, Yicheng Yang, Songsong Xie, Wumei Yuan, Yuanzhi Wang

**Affiliations:** 1 School of Medicine, Shihezi University, Shihezi, 832002 Xinjiang Uygur Autonomous Region China; 2 The First Affiliated Hospital, School of Medicine, Shihezi University, Shihezi, 832002 Xinjiang Uygur Autonomous Region China; 3 NHC Key Laboratory of Prevention and Treatment of Central Asia High Incidence Diseases, First Affiliated Hospital, School of Medicine, Shihezi University, Shihezi City, 832002 Xinjiang Uygur Autonomous Region China

**Keywords:** *Rickettsia raoultii*, Tacheng tick virus 1, Meningitis, Fever, Case report

## Abstract

**Background:**

Increasing numbers of tick-borne pathogens are being discovered, including those that infect humans. However, reports on co-infections caused by two or more tick-borne pathogens are scarce.

**Case presentation:**

A 38-year-old male farmer was bitten by a hard tick, presented with fever (37.7 °C), severe headache and ejection vomiting. Lumbar puncture was performed in the lateral decubitus. The cerebrospinal fluid (CSF) was clear, and analysis showed severe increased pressure (320 mm H_2_O), mild leukocytosis (126.0 × 10^6^/L, mononuclear cells accounting for 73%) and elevated total protein concentration (0.92 g/L). Bacterial cultures of CSF and blood were negative. The diagnosis of *Rickettsia raoultii* and Tacheng tick virus 1 (TcTV-1) co-infection was confirmed by amplifying four rickettsial genetic markers and the partial small (S) RNA segment of TcTV-1 from the patient’s blood. The patient gradually recovered after treatment with levofloxacin and ribavirin.

**Conclusions:**

This is the first reported co-infection case with fever and meningitis caused by *R*. *raoultii* and TcTV-1. It is vital to screen for multiple pathogens in tick-bitten patients, especially in those with severe complex symptoms.

## Background

Hard ticks (Acari: Ixodidae) are among the most versatile vectors, capable of transmitting several species of pathogens, including bacteria, protozoa, fungi, nematodes, and viruses, to humans, domestic and wildlife animals [1]. In Xinjiang Uygur Autonomous Region (XUAR, northwestern China), there are natural foci of multiple tick-borne diseases, such as spotted fever, Lyme borreliosis, Crimean-Congo hemorrhagic fever, and tick-borne encephalitis [2]. To date, ten spotted fever group *Rickettsia* (SFGR) were found in XUAR. Among these, *Rickettsia raoultii* was shown to be highly prevalent in northwestern China, i.e. 22.9% (404/1764) of ticks tested positive [3–6]. In previous studies, *R*. *raoultii* infections have also been increasingly detected in tick bite patients throughout China. Common nonspecific manifestations in 35 tick-bitten patients with mild to moderate or severe illness included fever (74.3%, 36.8–41.0 °C), malaise (71.4%), nausea (42.9%), myalgia (31.4%), lymphadenopathy (28.6%), vomiting (25.7%) and headache (14.3%). Only two patients (5.7%, 2/35) had meningeal syndrome [7–10].

In addition, some emerging bunyaviruses, such as Tacheng tick virus 1 (TcTV-1) and Tacheng tick virus 2 (TcTV-2), are suggested to caused human infections in XUAR [11, 12]. Epidemiological investigations showed that (i) 7.7% (26/339) of adult ticks tested positive for TcTV-1, and (ii) syndrome of an index patient infected with TcTV-1 included fever (as high as 39.0 °C), a local skin bulge (about 5 × 5 cm in size), rash, chill, muscle soreness and arthralgia. Moreover, the patient showed neurologic signs, such as headache and dizziness [11].

Previously, SFGR species were reported in co-infections with *Borreli burgdorferi*, *Anaplasma phagocytophilum*, *Orientia tsutsugamushi* and herpes simplex virus 2 [13–16]. In this study, we reported for the first time a tick bite patient co-infected with *R*. *raoultii* and TcTV-1 in XUAR.

## Case presentation

The patient was a 38-year-old previously healthy man, who was engaged in agricultural work. He reported that he was bitten by a tick on his left upper limb, at the tenth intercostal area of the left anterior axillary line on May 23, 2019. When he noticed and removed the tick, it was engorged. Two days later, a painful erythematous mass (about 6 × 6 mm in size) developed around the site of the tick bite. On May 30, he developed persistent fever (37.7 °C ± 0.2 ℃) and headache, and consulted a doctor at a local medical center. He was treated with calcium gluconate (1 g/day intravenously) and amoxicillin (0.5 g thrice daily orally) for 3 days. On June 4, his headache became increasingly severe, and was accompanied with nausea, mild neck stiffness and intense vomiting (more than 20 times per night). On June 5, the patient visited the Manasi County Hospital, XUAR. The patient received the brain MRI examination with DWI, and no obvious abnormality was found. Clinical hemogram, biochemical tests showed that most indexes were within the normal range. However, the white blood cell count (16.35 × 10^9^/L), absolute neutrophil count (13.25 × 10^9^/L), C-reactive protein (10.45 mg/L), glutamic-pyruvic transaminase (73.00 U/L), glutamic-oxalacetic transaminase (40.50 U/L) and total bile acid (21.3 U/L) levels. He was transferred under coma to the Department of Emergency Surgery, the First Affiliated Hospital, School of Medicine, Shihezi University. The cerebrospinal pressure of the patient reached 320 mm H_2_O (normal range, 80–180 mm H_2_O) as measured with lumbar puncture examination. The protein concentration of cerebrospinal fluid (CSF) increased to 0.92 g/L (normal range, 0.15–0.45 g/L). The leukocyte count was 126.0 × 10^6^/L (normal range, 0–8 × 10^6^/L), of which mononuclear cells accounted for 73.0%. Other clinical data are shown in Table 1. Blood and CSF samples were negative in BacT/Alert blood culture system.

**Table 1:** Clinical laboratory tests and complications of the patient

Laboratory findings, Signs			
Laboratory findings	Results (Hospital admission)	Results (Hospital discharged)	Normal range
Cerebrospinal fluid test			
Cerebrospinal pressure	320 mm H_2_O	220 mm H_2_O	80–180 mm H_2_O
Leukocyte count	126.0 × 10^6^/L	2.0 × 10^6^/L	0–8 × 10^6^/L
Protein concentration	0.92 g/L	0.56 g/L	0.15–0.45 g/L
Chloride	116 mmol/L	124 mmol/L	120–130 mmol/L
Pandy test	Positive	Weakly positive	Negative
Glucose	2.7 mmol/L	2.86 mmol/L	2.5–4.4 mmol/L
Monocytes account	73%	/	/
Cerebrospinal fluid culture	Negative	/	Negative
Hemogram			
A white blood cell count	16.35 × 10^9^/L	12.6 × 10^9^/L	4–10 × 10^9^/L
Neutrophil count	13.25 × 10^9^/L	8.41 × 10^9^/L	1.4–7 × 10^9^/L
Lymphocyte count	2.08 × 10^9^/L	3.1 × 10^9^/L	1.2–3.5 × 10^9^/L
Hemoglobin level	147 g/L	134 g/L	110–160 g/L
Platelet count	300 × 10^9^/L	326 × 10^9^/L	100–300 × 10^9^/L
Erythrocyte sedimentation rate (ESR)	24.00 mm/h	13 mm/h	0–15 mm/h
Blood biochemistry			
Albumin	43 g/L	43 g/L	40–55 g/L
Total bilirubin	22.1 umol/L	22.1 umol/L	2–28 umol/L
Glutamic-pyruvic transaminase (ALT)	73 u/L	41.0 u/L	0–40 u/L
Glutamic-oxaloacetic transaminase (AST)	40.5 u/L	18.0 u/L	0–40 u/L
Gamma-glutamyl transpeptidase (GGT)	127.0 u/L	63.0 u/L	12–43 u/L
Bacteriological examination			
Cerebrospinal fluid culture	Negative	/	Negative
Blood culture	Negative	/	Negative
Signs			
Fever			
Temperature on admission	37.8 ℃		
Highest temperature	37.9 ℃		
Complications			
A painful erythematous mass	Yes		
Headache	Yes		
Mild neck stiffness	Yes		
Nausea	Yes		
Vomiting	Yes		
Morbus asthenicus	Yes		
Poor diet and sleep	Yes		

“/” mean not detected

To search for more eventual co-infecting pathogens, the patient’ anticoagulated blood samples were collected upon admission (being diagnosed as acute meningitis). The DNA and RNA were extracted by a TIANamp Genomic DNA Kit and an TIANamp Genomic RNA Kit (Tiangen Biotech, Beijing, China), respectively. The complementary DNA (cDNA) was synthesized using the Revert Aid First Strand cDNA Synthesis Kit (Transgen Biotech, Beijing, China). Known tick-borne pathogens, including *Rickettsia* spp., *Anaplasma* spp., *Ehrlichia* spp., *Babesia* spp., *Francisella* spp., *Borrelia* spp., forest encephalitis virus, severe fever and thrombocytopenia syndrome virus, Crimean-Congo hemorrhagic fever virus, TcTV-1 and TcTV-2, were detected using nested PCR (nPCR) or reverse transcription-PCR (RT-PCR).

**Fig. 1:**
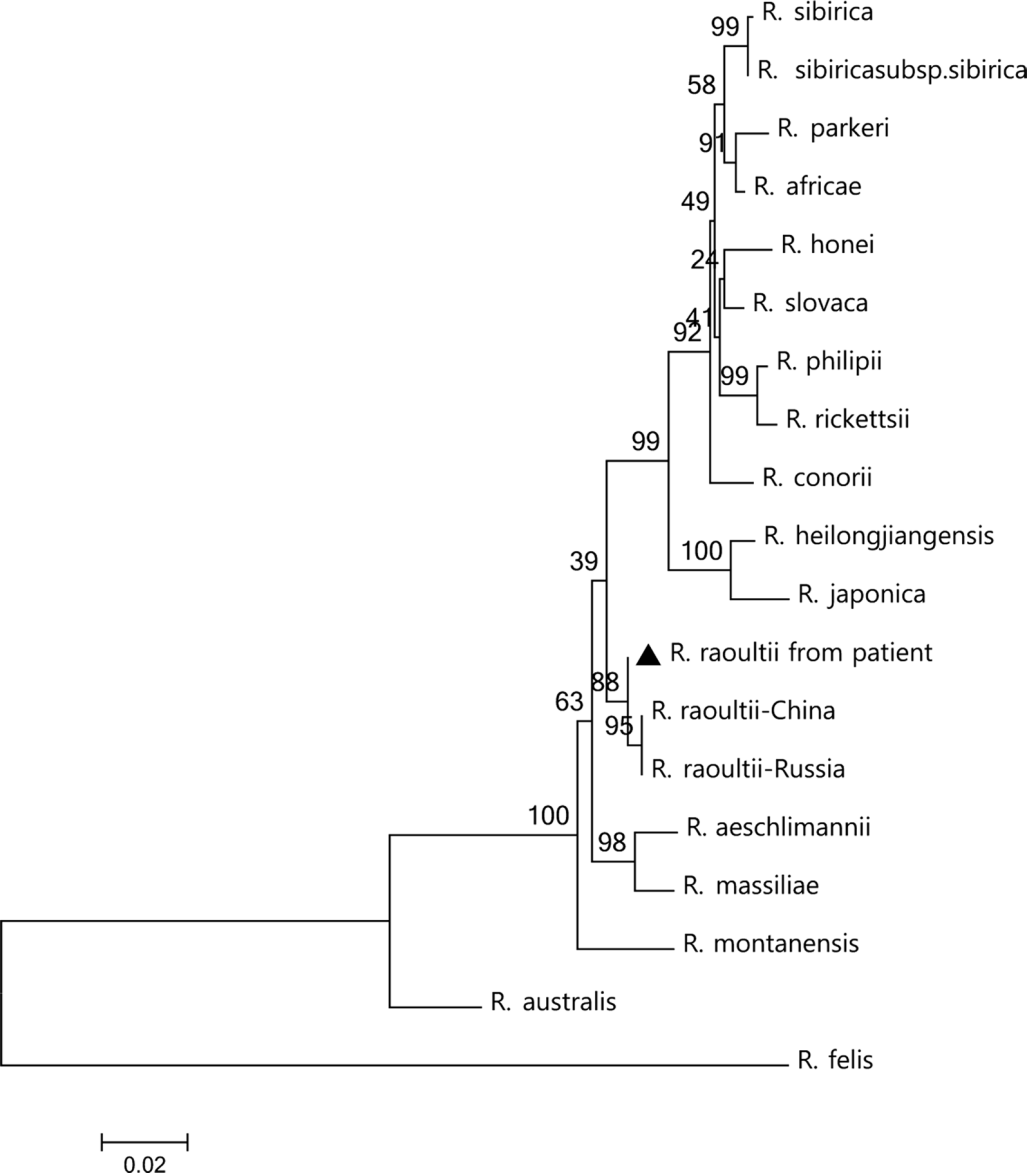
Phylogenetic tree of patient’s *R*. *raoultii* strain compared with avilable nucleotide sequences, selected using BLAST (http://blast.ncbi.nlm.nih.gov/Blast.cgi). The tree was constructed by maximum-likelihood (ML; 1000 bootstrap replicates) of concatenated sequence data of four genes (*17-kDa*-*sca*1-*rrs*-*omp*A) using Molecular Evolutionary Genetics Analysis (MEGA, version 7.0; http://www.megasoftware.net/). The concatenated sequence of *R*. *raoultii* are indicated by a black triangle (▲). The sequences of *Rickettsia felis* were used as the outgroup

**Fig. 2:**
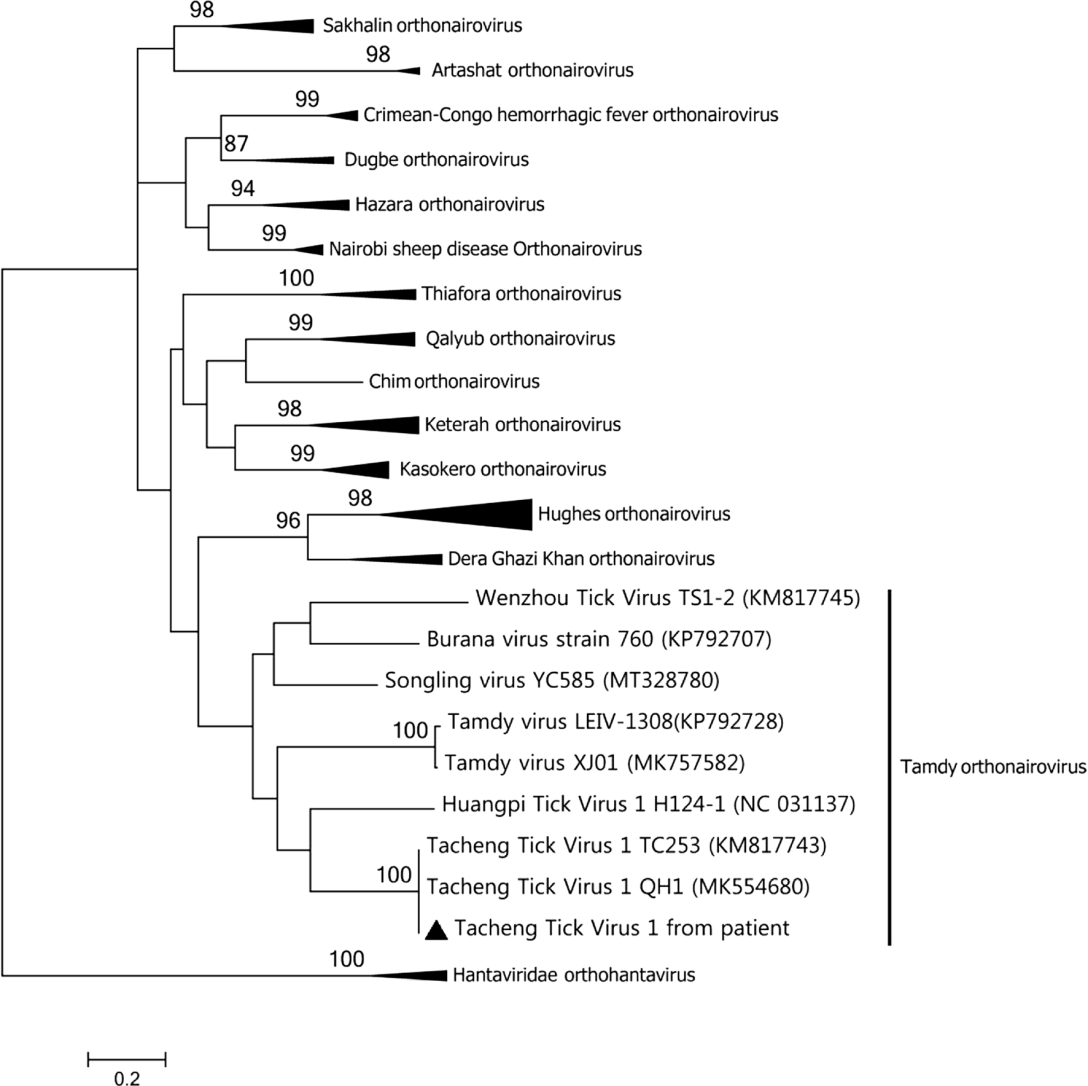
Phylogenetic tree of patient’s TcTV1 strain compared with available nucleotide sequences, selected using BLAST (http://blast.ncbi.nlm.nih.gov/Blast.cgi). Phylogenetic tree of TcTV1 and related orthonairovirus were constructed based on the partial sequences (328 bp) of nucleoprotein with maximum likelihood method (ML; 1000 bootstrap replicates) using Molecular Evolutionary Genetics Analysis (MEGA, version 7.0; http://www.megasoftware.net/). The TcTV1 sequence obtained in this study is indicated with a black triangle (▲). The sequences of hantaviridae orthohantavirus were used as the outgroup

Four *Rickettsia*-specific genetic markers, including 543-, 445-, 435-, and 364-bp products of the genes encoding the cell surface antigen 1 (sca1), outer membrane proteins A (ompA), 17 kilodalton antigen (17-kDa), and mitochondrial 16S ribosomal DNA (16SrDNA), were amplified using previously described primers [17]. At the same time, the partial S segment of TcTV-1 (328 bp) was also screened by RT-PCR [11]. The PCR products were purified using the TIAN gel Midi Purification Kit (Tiangen, Beijing, China) and sequenced by Sanger di-deoxy sequencing method (Sangon Biotech, Shanghai, China). Each test was repeated three times. Obtained sequences corresponded to those of *R*. *raoultii* and TcTV-1 as revealed by BLAST search (http://www.ncbi.nlm.nih.gov/BLAST/). Phylogenetic trees were constructed using the Maximum-Likelihood method in MEGA 7.0 software (Figs. 1 and 2) [18]. The samples tested negative for other tick-borne pathogens. All sequences obtained in this study were deposited in the GenBank database [TcTV-1: MW752511; *ompA*: MW752512; *sca1*: MW752513; *17-kDa*: MW752514; *16SrDNA*: MW752515;].

The patient was treated with intravenous levofloxacin (0.5 g/day) and ribavirin (0.5 g/day) for 8 days and supportive treatment (potassium chloride oral solution, intravenous 20% mannitol, vitamin C and B6 injections). His mental status and body temperature improved at day 3 during hospitalization. On June 15, the patient was discharged from hospital although the data of mild laboratory abnormalities persisted, including the elevated protein concentration of CSF and gamma-glutamyl transpeptidase levels of serum. The patient was put on continuous oral doxycycline (100 mg, twice daily) and ribavirin (0.45 g, thrice daily) treatment at home for 4 days. After a 14-month follow-up, the tick bite patient recovered with no other complications.

## Discussion and conclusions

In this study, we reported a severe acute meningitis case in a tick bite patient co-infected with *R. raoultii* and TcTV-1. Clinical manifestations included fever (37.7 °C ± 0.2 ℃), severe headache, persistently intense vomiting, mild neck stiffness and raised intracranial pressure (320 mm H_2_O) with mild mononuclear cells and elevated CSF protein levels. It is well known that spotted fever can cause central nervous system infection, presenting as fever, headache, vomiting, increased intracranial pressure and aseptic meningitis [19–21]. In similar studies, slightly increased intracranial pressure (200 mm H_2_O) was reported in a case of neurologic abnormalities caused by *R*. *raoultii* in northwestern China after tick bite, resulting in right eyelid ptosis, lethargy, fever (38.0–41.0 °C), headache and CSF leukocytosis [9]. Elevated intracranial pressure (235 mm H_2_O) have been reported in a case of Japanese spotted fever with central nervous system involvement and multiple organ failure [22]. In the present case, severe increased intracranial pressure (320 mm H_2_O) was also present, complicated with persistently intense vomiting. This finding means that (i) CSF pressure measurement is necessary in spotted fever patients with neurologic signs, and (ii) some emerging tick-borne viruses should also be considered as co-infecting agents in all endemic areas of spotted fever.

Previously, an index patient single-infected with TcTV-1 also showed lymphocytic meningitis similar to that after SFGR infection [9, 11, 22]. In our study, the patient co-infected with TcTV-1 and *R*. *raoultii* presented increased intracranial pressure (320 mm H_2_O), resulting in severe headache and intense vomiting (> 20 times per night), which might account for overlapping clinical signs of the central nervous system between TcTV-1 and *R*. *raoultii* infections. In addition, there are major differences in therapeutic measures between infections caused by various tick-borne pathogens, as exemplified by *R*. *raoultii* and TcTV-1. Therefore, early diagnosis based on identification of multiple pathogens, is crucial in tick-bitten patients with severe clinical syndrome. Public health workers and physicians need to be more aware of the clinical complexity of tick-borne pathogen infections, especially in high-risk areas of tick-borne diseases.

There are several limitations to our study. Firstly, rickettsia and TcTV-1 markers should have also been detected in CSF samples, although cellular and biochemical characteristics of CSF were useful in early diagnosis. Secondly, bacterial and viral metagenomic analysis should have been performed in more clinical samples (e.g., pharyngeal swab, urine, and cerebrospinal fluid) associated with the tick-bite patients. In future cases, these would help us to better understand complex clinical manifestations due to infection of single or multiple tick-borne pathogens.

## Data Availability

The datasets generated and analysed during the current study are available in the GenBank repository, Accession number to datasets: TcTV-1: MW752511; *omp*A: MW752512; *sca*1: MW752513; *17-kDa*: MW752514; *16SrDNA*: MW752515.

## Abbreviations

XUAR: Xinjiang Uygur Autonomous Region
SFGR: Spotted fever group Rickettsia
*16S rDNA*: Mitochondrial 16S ribosomal DNA
*17-kDa*: 17 Kilodalton antigen
*omp*A: Outer membrane proteins A
*sca*1: Cell surface antigen 1
CSF: Cerebrospinal fluid
MEGA7.0: Molecular Evolutionary Genetics Analysis 7.0
PCR: Polymerase chain reaction
*R*. *raoultii*: *Rickettsia raoultii*
TcTV-1: Tacheng tick virus 1
